# The association between dietary acid load and body composition in physical education students aged 18–25 years

**DOI:** 10.1186/s41043-022-00340-8

**Published:** 2022-12-19

**Authors:** Maryam Mansordehghan, Elnaz Daneshzad, Vahid Basirat, Bahram Pourghassem Gargari, Tohid Rouzitalab

**Affiliations:** 1 Department of Nutritional Sciences, School of Nutritional Sciences and Food Technology, Kermanshah University of Medical Sciences, Kermanshah, Iran; 2 Non-Communicable Diseases Research Center, Alborz University of Medical Sciences, Karaj, Iran; 3 Department of Gastroenterology, School of Medicine, Isfahan University of Medical Sciences and Health Services, Isfahan, Iran; 4 Department of Biochemistry and Diet Therapy, Nutrition Research Center, School of Nutrition and Food Sciences, Tabriz University of Medical Sciences, Tabriz, Iran

**Keywords:** Dietary acid load, Body composition, Body mass index, Waist circumference, Fat-free mass, Muscle mass, Body fat

## Abstract

**Aim:**

To find the association between dietary acid load (DAL) and body composition in physical education students.

**Methods:**

This study was carried out on 207 students of both genders aged 18–25 years. DAL was calculated based on potential renal acid load (PRAL) and net endogenous acid production (NEAP) methods. Anthropometric indices were measured. Bioelectric impedance was used to assess body composition and other related items.

**Results:**

The mean score of NEAP and PRAL was 80.18 ± 31.30 and 33.94 ± 22.11, respectively. The mean weight and fat mass of subjects were 64.05 ± 9.72 kg and 20.28 ± 0.67 kg, respectively. Participants in the highest tertile of PRAL had a higher weight (64.56 ± 1.14 kg) in comparison with participants in the lowest tertile (61.65 ± 1.19 kg) (*P* = 0.027). After adjusting for confounders, a significant positive association was found between NEAP score and hip circumference (*β* = 0.206, *P* = 0.039), body mass index (*β* = 0.214, *P* = 0.031), fat mass (*β* = 0.218, *P* = 0.001) and body adiposity index (*β* = 0.182, *P* = 0.037). Furthermore, a statistically significant negative association was observed between total body water and NEAP score (*β* = − 0.217, *P* = 0.001) and the percentage of fat-free mass and NEAP (*β* = − 0.229, *P* = 0.001).

**Conclusion:**

Individuals with a higher DAL score may have a higher weight, fat mass and hip circumference and a lower fat-free mass. In addition, there might be an association between DAL and obesity.

## Introduction

Abnormal and excessive fat accumulation is a well-known contributing factor to chronic diseases such as diabetes, cancer, hypertension, and other health problems. The higher prevalence of overweight and obesity is related to health disturbances and disabilities that impose a higher financial burden on the health system [1]. The role of nutrition and diet therapy in controlling obesity is well established. Diet compositions can alter the acid–base balance [2]. Previous studies have shown that metabolic acidosis may be related to obesity. The previous evidence shows that adherence to an acidogenic diet leads to the accumulation of hydrogen ions related to weight gain [3]. The higher intake of animal-based foods, meats, and western dietary patterns leads to higher organic acid production and fatty acid oxidation, particularly in obese individuals [3]. It has been reported that western dietary patterns with a higher intake of meats, eggs and refined grains and a higher dietary acid load (DAL) may induce central obesity and metabolic syndrome [4, 5]. Li et al. demonstrated that a higher DAL is attributed to the adipogenic effects [6]. Furthermore, acidosis leads to lower protein synthesis, higher proteolysis and amino acid oxidation which causes a greater loss of muscle mass [7]. In addition, metabolic acidosis invigorates cortisol production and is related to metabolic disturbances [8]. However, the evidence from associations between DAL and anthropometric indices and body composition is inconsistent [9–11]. A meta-analysis has shown that DAL was associated with a higher obesity prevalence [1], and DAL reduction may help the prevention of obesity and other related disorders. Additionally, a study revealed a significant positive association between an alkalogenic diet and fat-free mass [12]. The established methods to assess the dietary acid load are net endogenous acid production (NEAP) and potential renal acid load (PRAL) [13, 14]. These scores are correlated with 24-h urinary acid load and have been validated in adults [13].

Acid–base balance is an important factor for health status and physical performance. Acids production increase with physical activity and a higher basal metabolic rate. Physical activity and sports lead to changes in the acid–base balance and produce additional organic acids in the body [15]. Therefore, more active individuals might have more organic acids in the body and metabolic disturbances. To the best of our knowledge, no study has examined associations between DAL and body composition in Physical Education students who are physically active. The present study investigates the association between DAL and body composition and anthropometric indices in physical education students aged 18–25 years. DAL was measured using NEAP and PRAL scores.

## Methods

In this cross-sectional study, 207 healthy men and women Physical Education Faculty of Tabriz University in Tabriz, Iran, were randomly recruited between June and July 2013. The sample size for the present study was calculated by G-power software regarding the relationship between DAL and weight as the effect size of interest, considering 95% confidence and 80% power by a two-tailed test (*N* = 207). By random sampling method, 207 physical education students aged 18–25 years were recruited. All subjects signed the written consent form before participating in this study. The inclusion criteria included an age range of 18 to 25 years old and being a student of physical education for at least 6 months. Individuals with chronic diseases including cancers, thyroid problems, cardiovascular and kidney disease were excluded. Students who reported a total energy intake of < 800 and > 4200 kcal were excluded as the under and over reports of energy intakes [16]. The present study was carried out based on the recommendations of the ethics committee of Tabriz University of Medical Sciences (ethical code: 5/4/2357; 11/6/2013).

Socio-demographic characteristics, including age, gender, family income, parents' occupation, homeownership, the number of essential items for living, smoking habits, medical history, and supplement consumption, were collected using a questionnaire. As a confounder, socioeconomic status (SES) was assessed using a Likert scale for each socioeconomic item, including parents' occupation and income, homeownership, and the number of essential items for living. Finally, all scores are summed up to measure the SES. Physical activity level was recorded using a self-reported questionnaire and expressed as a metabolic equivalent hours/day. The scale ranged from sleep/rest (0.9) to high-intensity physical activity (< 6) [17].

### Assessment of anthropometric measures and body composition

The body weight was measured to the nearest 0.1 kg while participants were in light clothes and without shoes using a calibrated electronic Seca scale (Seca 769, Germany). At the beginning of the day of data collection, the scale was calibrated against 100 kg weight. Height was measured in the stand position with shoulders in a normal position and without shoes, using a stadiometer to the nearest 0.1 cm (Seca 220, Germany). Body mass index (BMI) was calculated as weight divided by height (kg/m^2^). Waist circumference (WC) was measured at 0.1 cm using an unstretched tape measure at the midpoint between the lower costal border and above the iliac crest at the end of normal expiration (Seca 201, Germany). Hip circumference (HC) was measured at the maximum perimeter using a non-stretchable tape to the nearest 0.1 cm (Seca 201, Germany). Waist-to-hip ratio (WHR) was calculated by dividing the waist by hip size. Waist-to-height ratio (WHtR) was calculated by dividing WC (cm) by height (cm). Bioelectric impedance using an auto-calibrated in body 230 (Biospace, Dogok-dong, South Korea) was used for the assessment of body composition components, including muscle mass (MM), fat mass (FM), fat-free mass (FFM), total body water (TBW), and body adiposity index (BAI) and their percentages while participants were in light clothes. Regarding the standard conditions [18], subjects were 2-h fasting before measuring the body composition with a lack of intense activity 12 h before the test, were not in their menstrual periods, and were in light clothes. All the data collection was performed by an expert nutritionist. Also, all anthropometric indices were treated as a continuous variable.

### Assessment of dietary intake

A validated semiquantitative food frequency questionnaire (FFQ 168-item) was used to record participants' dietary intakes over the past year [19]. All participants filled in the frequency and amount of consumption of each food item on a daily, weekly, or monthly over the past year. The portion sizes of all reported foods were converted to grams/day using household measurements [20]. Nutritionist IV software (Version 7.0; N-Squared Computing, Salem, OR, USA) adapted for Iranian foods was used for nutrient analysis [21].

### Assessment of dietary acid load (DAL)

The dietary acid load was calculated according to the potential renal acid load (PRAL) [22] and the net endogenous acid production (NEAP) methods [14].

$$\begin{aligned} & {\text{PRAL}}\left( {{\text{milliequivalents}}\;{\text{of}}\;{\text{acid}}\;{\text{per}}\;{\text{day}};\;{\text{mEq}}/{\text{d}}} \right) = \left( {{\text{protein}}\left[ {{\text{g}}/{\text{d}}} \right] \times 0.{49}} \right) \\ & \quad + \left( {{\text{Phosphorus}}\left[ {{\text{mg}}/{\text{d}}} \right] \times 0.0{37}} \right) {-}\left( {{\text{Potassium}}\left[ {{\text{mg}}/{\text{d}}} \right] \times 0.0{21}} \right) \\ & \quad {-}\left( {{\text{Calcium}}\left[ {{\text{mg}}/{\text{d}}} \right] \times 0.0{13}} \right){-}\left( {{\text{Magnesium}}\left[ {{\text{mg}}/{\text{d}}} \right] \times 0.0{26}} \right) \\ \end{aligned}$$

$$\begin{aligned} &{\text{Estimated NEAP}};\\ &{\text{ mEq}}/{\text{d}}\, = \,\left( {{54}.{5}\, \times \,{\text{protein intake }}\left[ {{\text{g}}/{\text{d}}} \right]/{\text{potassium intake }}\left[ {{\text{mEq}}/{\text{d}}} \right]} \right) - {1}0.{2}.\end{aligned}$$

Two scores were calculated for every participant using these two formulas (one for NEAP and one for PRAL). In the end, both scores were categorized into tertiles to have a better comparison between the lowest and the highest DAL score.

### Statistical analysis

The normality distribution of variables was assessed using the Kolmogorov–Smirnov test. To compare participants, they were categorized into tertiles of DAL scores. Participants' characteristics were compared using ANOVA or chi-square tests against the NEAP and PRAL tertiles for continuous and categorical variables, respectively, and reported as the mean ± standard deviation (SD) or percentage. Dietary intakes according to the tertiles of DAL were assessed using ANCOVA, controlling for calorie intake. Furthermore, anthropometric indices and body compositions were examined over DAL tertiles using ANCOVA in crude and adjusted models. Multivariate linear regression analysis was used to examine the association between anthropometric indices (numerical) and body composition, and DAL scores. In the adjusted model, the analysis was adjusted for the confounders including age, gender, PA, SES, calorie, protein, and fat intake. Data analysis was carried out with SPSS ver.16 (Chicago, IL), and *P* value < 0.05 was considered as statistically significant.

## Results

The mean score of NEAP and PRAL was 80.18 ± 31.30 and 33.94 ± 22.11, respectively. Table 1 presents the general characteristics of the participants. The mean age and weight of subjects were 21.09 years and 64.05 kg, respectively. Regarding the socioeconomic status, 22.7% of participants were poor, 54.6% were considered moderate, and 22.7% were categorized as rich. There was a statistically significant association between PA and tertiles of DAL (*P* < 0.0001). Also, a significant association was observed between weight and tertiles of PRAL (*P* = 0.045). There was no significant association between SES and tertiles of DAL. Moreover, there were no statistically significant differences between tertiles of DAL and other general characteristics.

**Table 1:** Characteristics of participants among tertiles of dietary acid load

Variables	Total	Tertiles of NEAP			P value^*^	Tertiles of PRAL			P value^*^
		1	2	3		1	2	3	
		< 61.41	61.41–88.24	> 88.24		< 23.46	23.46–44.79	> 44.79	
Number	207	69	69	69		69	69	69	
Age (Year)	21.09 ± 1.67	21.08 ± 1.55	20.92 ± 1.81	21.27 ± 1.64	0.477	21.10 ± 1.69	20.84 ± 1.70	21.34 ± 1.61	0.206
Weight (kg)	64.05 ± 9.72	63.08 ± 8.85	64.76 ± 9.85	64.31 ± 10.46	0.581	61.69 ± 7.60	65.69 ± 10.9	64.77 ± 9.95	0.040"*"
Height (cm)	170.26 ± 9.18	170.18 ± 8.66	170.81 ± 9.44	169.79 ± 9.50	0.809	168.36 ± 8.72	170.71 ± 9.69	171.72 ± 8.89	0.087
BMI (kg/m^2^)	22.04 ± 2.52	21.75 ± 2.32	22.16 ± 2.63	22.22 ± 2.60	0.485	21.77 ± 2.30	22.48 ± 2.87	21.88 ± 2.33	0.211
PA (met.h/d)	46.04 ± 5.62	43.06 ± 3.83	46.01 ± 5.71	49.06 ± 5.48	< 0.0001"*"	44.24 ± 4.79	45.15 ± 5.24	48.75 ± 5.80	< 0.0001"*"
Gender n (%)					0.209				0.499
Female	102 (49.3)	31 (30.4)	31 (30.4)	40 (39.2)		36 (35.3)	36 (35.3)	30 (29.4)	
SES n (%)					0.917				0.432
Poor	47 (22.7)	17 (36.2)	15 (31.9)	15 (31.9)		17 (36.2)	18 (38.3)	12 (25.5)	
Moderate	113 (54.6)	37 (32.7)	36 (31.9)	40 (35.4)		33 (29.2)	39 (34.5)	41 (36.3)	
Rich	47 (22.7)	15 (31.9)	18 (38.3)	14 (29.8)		19 (40.4)	12 (25.5)	16 (34.0)	
SC n (%)					0.710				0.220
NO	184 (88.9)	60 (32.6)	63 (34.2)	61 (33.2)		60 (32.6)	65 (35.3)	59 (32.1)	

*NEAP*: net endogenous acid production, *PRAL* potential renal acid load, *BMI* body mass index, *PA* physical activity, *SES* socioeconomic status, *SC* supplement consumption

^*^Calculated by chi-square (presented as number and percent) and ANOVA (presented as mean ± SD) for qualitative and quantitative variables, respectively

“*”Significant p values

Participants’ daily dietary intakes are presented in Table 2. The consumption of protein and fat was higher in the third tertile of both DAL indices (< 0.05). Intake of energy and potassium was lower in the third tertile of both DAL indices (< 0.0001). Intake of calcium and phosphorus was lower in the third tertile of both indices of DAL (< 0.0001).

**Table 2:** Daily dietary intakes of participants among tertiles of dietary acid load

Variables	Total	Tertiles of NEAP			P value^*^	Tertiles of PRAL			P value^*^
		1	2	3		1	2	3	
		< 61.41	61.41–88.24	> 88.24		< 23.46	23.46–44.79	> 44.79	
Number	207	69	69	69		69	69	69	
Energy (kcal)	2370.19 ± 28.09	2498.49 ± 48.23	2423.64 ± 52.03	2188.44 ± 36.99	< 0.0001"*"	2400.03 ± 51.59	2499.53 ± 46.99	2211.01 ± 40.87	< 0.0001"*"
Protein (g)	112.76 ± 1.77	90.38 ± 2.39	119.98 ± 2.35	127.92 ± 2.43	< 0.0001"*"	88.89 ± 2.09	116.87 ± 2.13	132.52 ± 2.15	< 0.0001"*"
CHO (g)	279.61 ± 5.64	289.91 ± 8.78	276.69 ± 8.64	272.23 ± 8.94	0.351	292.52 ± 8.60	275.47 ± 8.75	270.84 ± 8.83	0.177
Fat (g)	92.15 ± 2.04	84.93 ± 2.81	98.16 ± 2.77	93.35 ± 2.87	0.004"*"	82.62 ± 2.69	92.58 ± 2.74	101.24 ± 2.77	< 0.0001"*"
Potassium (mg)	2841.82 ± 51.74	3273.97 ± 54.33	3006.70 ± 53.51	2244.80 ± 55.32	< 0.0001"*"	3050.61 ± 69.36	2900.57 ± 70.58	2574.30 ± 71.23	< 0.0001"*"
Calcium (mg)	1202.81 ± 26.15	1145.16 ± 46.09	1243.08 ± 45.39	1220.18 ± 46.93	0.290	989.80 ± 40.79	1213.31 ± 41.50	1405.31 ± 41.89	< 0.0001"*"
Magnesium (mg)	277.18 ± 5.36	267.20 ± 9.46	275.57 ± 9.32	288.78 ± 9.64	0.295	279.48 ± 9.25	261.07 ± 9.41	291.01 ± 9.50	0.088
Phosphorus (mg)	1654.23 ± 30.06	1578.43 ± 52.62	1707.69 ± 51.82	1676.56 ± 53.58	0.193	1358.30 ± 42.90	1638.20 ± 43.65	1966.18 ± 44.06	< 0.0001"*"

*NEAP* net endogenous acid production, *PRAL* potential renal acid load, *CHO* carbohydrate

^*^Calculated by one-way ANOVA test for energy intake and ANCOVA test for other variables (adjusted for energy intake)

“*”Significant p values

Data presented as mean ± standard error

Table 3 shows the distribution of anthropometric indices over NEAP and PRAL tertiles. Based on the results, subjects who were in the highest tertile of PRAL had a higher weight (64.56 ± 1.14 kg) in comparison with participants in the lowest tertile (61.65 ± 1.19 kg) (*P* = 0.027). Also, subjects in the highest tertile of NEAP had a higher hip circumference (*P* = 0.037) and a higher body fat (*P* = 0.031). Participants in the highest tertile of NEAP had a lower percentage of TBW (*P* = 0.03) and FFM (*P* = 0.018). Furthermore, there was a significant association between NEAP and BAI (*P* = 0.025).

**Table 3:** Anthropometric indices among tertiles of dietary acid load

Variables	Total	Tertiles of NEAP			P value^*^	Tertiles of PRAL			P value^*^
		1	2	3		1	2	3	
		< 61.41	61.41–88.24	> 88.24		< 23.46	23.46–44.79	> 44.79	
Number	207	69	69	69		69	69	69	
Weight (kg)	64.05 ± 0.67								
Crude model		63.08 ± 1.06	64.76 ± 1.18	64.31 ± 1.25	0.581	61.69 ± 0.91	65.69 ± 1.32	64.77 ± 1.19	0.581
Adjusted model		62.07 ± 1.18	64.16 ± 0.95	65.92 ± 1.10	0.121	61.65 ± 1.19	65.94 ± 0.95	64.56 ± 1.14	0.027
WC (cm)	75.54 ± 0.56								
Crude model		75.50 ± 1.02	76.08 ± 0.89	75.02 ± 1.01	0.747	74.81 ± 0.83	76.31 ± 1.13	75.49 ± 0.94	0.747
Adjusted model		74.92 ± 1.05	75.61 ± 0.84	76.08 ± 0.98	0.784	75.43 ± 1.07	76.34 ± 0.84	74.84 ± 1.01	0.473
HC (cm)	93.93 ± 0.41								
Crude model		92.84 ± 0.71	94.51 ± 0.73	94.42 ± 0.70	0.183	93.29 ± 0.70	94.79 ± 0.78	93.69 ± 0.66	0.183
Adjusted model		91.87 ± 0.89	94.62 ± 0.71	95.29 ± 0.82	0.037	93.28 ± 0.91	94.63 ± 0.72	93.87 ± 0.87	0.490
WHR	0.80 ± 0.004								
Crude model		0.80 ± 0.008	0.80 ± 0.007	0.79 ± 0.007	0.347	0.79 ± 0.007	0.80 ± 0.009	0.80 ± 0.007	0.347
Adjusted model		0.81 ± 0.008	0.79 ± 0.006	0.79 ± 0.007	0.426	0.80 ± 0.008	0.80 ± 0.006	0.79 ± 0.008	0.575
WHtR	0.44 ± 0.002								
Crude model		0.44 ± 0.005	0.44 ± 0.004	0.44 ± 0.005	0.863	0.44 ± 0.005	0.44 ± 0.004	0.44 ± 0.005	0.863
Adjusted model		0.43 ± 0.006	0.44 ± 0.005	0.44 ± 0.006	0.731	0.44 ± 0.007	0.44 ± 0.005	0.43 ± 0.006	0.659
BMI (kg/m^2^)	22.04 ± 0.17								
Crude model		21.75 ± 0.28	22.16 ± 0.31	22.22 ± 0.31	0.485	21.75 ± 0.28	22.16 ± 0.31	22.22 ± 0.31	0.485
Adjusted model		21.26 ± 0.37	22.21 ± 0.30	22.66 ± 0.34	0.059	21.59 ± 0.38	22.40 ± 0.30	22.14 ± 0.36	0.269
Muscle Mass (kg)	28.76 ± 0.47								
Crude model		28.45 ± 0.77	29.60 ± 0.81	28.23 ± 0.88	0.448	27.27 ± 0.74	29.12 ± 0.88	29.89 ± 0.81	0.448
Adjusted model		28.40 ± 0.58	28.98 ± 0.47	28.90 ± 0.54	0.770	27.68 ± 0.58	29.50 ± 0.46	29.10 ± 0.56	0.067
Fat Mass (kg)	20.28 ± 0.67								
Crude model		19.91 ± 1.15	18.99 ± 1.14	21.94 ± 1.18	0.185	19.91 ± 1.15	18.99 ± 1.14	21.94 ± 1.18	0.185
Adjusted model		18.61 ± 0.97	19.85 ± 0.78	22.38 ± 0.90	0.031	12.11 ± 0.78	13.40 ± 0.62	12.78 ± 0.75	0.431
Fat Mass (%)	20.28 ± 0.67								
Crude model		19.91 ± 1.15	18.99 ± 1.14	21.94 ± 1.18	0.185	21.22 ± 1.23	21.29 ± 1.22	18.33 ± 1.00	0.185
Adjusted model		18.61 ± 0.97	19.85 ± 0.78	22.38 ± 0.90	0.031	20.10 ± 1.00	20.63 ± 0.79	20.12 ± 0.95	0.872
TBW (kg)	37.59 ± 0.56								
Crude model		37.19 ± 0.92	38.59 ± 0.96	36.99 ± 1.05	0.457	35.76 ± 0.88	38.06 ± 1.05	38.95 ± 0.97	0.457
Adjusted model		37.19 ± 0.69	37.82 ± 0.56	37.75 ± 0.64	0.799	36.29 ± 0.70	38.53 ± 0.55	37.95 ± 0.66	0.057
TBW (%)	58.43 ± 0.49								
Crude model		58.69 ± 0.84	59.41 ± 0.84	57.19 ± 0.87	0.176	57.72 ± 0.90	57.70 ± 0.90	59.86 ± 0.74	0.176
Adjusted model		59.63 ± 0.71	58.78 ± 0.57	56.88 ± 0.66	0.030	58.53 ± 0.73	58.21 ± 0.58	58.55 ± 0.70	0.901
FFM (kg)	51.34 ± 0.76								
Crude model		50.93 ± 1.24	52.61 ± 1.31	50.47 ± 1.43	0.491	48.99 ± 1.19	51.90 ± 1.44	53.12 ± 1.32	0.491
Adjusted model		50.93 ± 0.95	51.59 ± 0.76	51.49 ± 0.88	0.879	49.70 ± 0.96	52.52 ± 0.76	51.79 ± 0.91	0.087
FFM (%)	79.80 ± 0.66								
Crude model		80.37 ± 1.11	80.99 ± 1.13	78.02 ± 1.18	0.158	79.06 ± 1.20	78.69 ± 1.22	81.64 ± 1.00	0.158
Adjusted model		81.44 ± 0.95	80.16 ± 0.76	77.58 ± 0.88	0.018	80.14 ± 0.99	79.36 ± 0.78	79.89 ± 0.94	0.792
BAI	0.55 ± 0.002								
Crude model		0.54 ± 0.005	0.55 ± 0.005	0.55 ± 0.005	0.305	0.54 ± 0.005	0.55 ± 0.005	0.55 ± 0.005	0.305
Adjusted model		0.53 ± 0.006	0.55 ± 0.004	0.56 ± 0.005	0.025	0.55 ± 0.006	0.55 ± 0.005	0.55 ± 0.005	0.968

*NEAP* net endogenous acid production, *PRAL* potential renal acid load, *WC* waist circumference, *HC* hip circumference, *BMI* body mass index; *TBW* total body water; *FFM* fat-free mass, *WHR* waist-to-hip ratio, *WHtR* waist-to-hip ratio, *BAI* body adiposity index

^*^Crude and adjusted model models are calculated by one-way ANOVA and ANCOVA tests, respectively. The adjusted model is adjusted for age, gender, physical activity, socioeconomic status, and energy, protein, and fat intakes

Data presented as mean ± standard error

Table 4 demonstrates the association between anthropometric indices and the score of NEAP and PRAL in the crude and adjusted models. There was a significant positive association between weight and PRAL in the crude model. However, after adjusting for confounders, this significant association disappeared. Also, there was a significant positive association between PRAL and muscle mass and TBW and FFM in the crude model; however, these significant associations disappeared after controlling for age, sex, SES, PA, and intake of energy, fat, and protein.

**Table 4:** Association of anthropometric indices and dietary acid load using linear regression

Variables	NEAP score			PRAL score		
	β	95% CI	P value	β	95% CI	P value
Weight (kg)						
Crude model	0.016	(− 0.03, 0.04)	0.821	0.144	(0.003, 0.12)	0.039
Adjusted model	0.153	(− 0.002, 0.09)	0.060	0.155	(− 0.01, 0.14)	0.089
WC (cm)						
Crude model	− 0.043	(− 0.47, 0.02)	0.543	0.060	(− 0.02, 0.07)	0.389
Adjusted model	0.075	(− 0.02, 0.06)	0.383	0.017	(− 0.06, 0.07)	0.856
HC (cm)						
Crude model	0.088	(− 0.01, 0.04)	0.206	0.049	(− 0.02, 0.05)	0.485
Adjusted model	0.206	(0.002, 0.07)	0.039	0.085	(− 0.03, 0.08)	0.499
WHR						
Crude model	− 0.109	(− 0.01, 0.01)	0.117	0.056	(− 0.1, 0.01)	0.419
Adjusted model	− 0.049	(− 0.01, 0.001)	0.536	− 0.038	(− 0.01, 0.01)	0.667
WHtR						
Crude model	− 0.023	(− 0.01, 0.01)	0.745	− 0.022	(− 0.01, 0.01)	0.756
Adjusted model	0.089	(− 0.1, 0.01)	0.378	− 0.012	(− 0.01, 0.001)	0.915
BMI (kg/m^2^)						
Crude model	0.062	(− 0.006, 0.01)	0.377	0.041	(− 0.01, 0.02)	0.553
Adjusted model	0.214	(0.002, 0.03)	0.031	0.158	(− 0.007, 0.04)	0.154
Muscle mass (kg)						
Crude model	− 0.064	(− 0.04, 0.01)	0.362	0.155	(0.006, 0.09)	0.026
Adjusted model	− 0.009	(− 0.02, 0.02)	0.877	0.067	(− 0.01, 0.05)	0.286
Fat mass (kg)						
Crude model	0.132	(− 0.001, 0.08)	0.058	− 0.099	(− 0.10, 0.01)	0.155
Adjusted model	0.218	(0.02, 0.10)	0.001	0.083	(− 0.02, 0.10)	0.272
Fat mass (%)						
Crude model	0.132	(− 0.001, 0.08)	0.058	− 0.099	(− 0.10, 0.01)	0.155
Adjusted model	0.218	(0.02, 0.10)	0.001	0.083	(− 0.02, 0.10)	0.272
TBW (kg)						
Crude model	− 0.060	(− 0.05, 0.02)	0.389	0.158	(0.008, 0.10)	0.023
Adjusted model	− 0.010	(− 0.03, 0.02)	0.860	0.064	(− 0.02, 0.06)	0.314
TBW (%)						
Crude model	− 0.132	(− 0.001, 0.08)	0.058	0.099	(− 0.01, 0.07)	0.156
Adjusted model	− 0.217	(− 0.07, − 0.02)	0.001	− 0.082	(− 0.07, 0.02)	0.274
FFM (kg)						
Crude model	− 0.066	(− 0.07, 0.02)	0.342	0.151	(0.007, 0.14)	0.030
Adjusted model	− 0.015	(− 0.04, 0.03)	0.795	0.058	(− 0.03, 0.09)	0.362
FFM (%)						
Crude model	− 0.145	(− 0.08, − 0.01)	0.037	0.086	(− 0.02, 0.09)	0.215
Adjusted model	− 0.229	(− 0.11, − 0.03)	0.001	− 0.094	(− 0.10, 0.02)	0.213
BAI						
Crude model	0.109	(− 0.01, 0.01)	0.116	− 0.063	(− 0.01, 0.01)	0.367
Adjusted model	0.182	(− 0.01, 0.001)	0.037	0.043	(− 0.001, 0.01)	0.663

*NEAP* net endogenous acid production, *PRAL* potential renal acid load, *WC* waist circumference, *HC* hip circumference, *BMI* body mass index; *TBW* total body water; *FFM* fat-free mass, *WHR* waist-to-hip ratio, *WHtR* waist-to-hip ratio, *BAI* body adiposity index

^*^P values are calculated linear regression. The adjusted model is adjusted for age, gender, physical activity, socioeconomic status, and energy, protein, and fat intakes

On the other hand, adjusting for confounders resulted in a significant positive association between the hip circumference and NEAP (*β* = 0.206, *P* = 0.039) and BMI and NEAP (*β* = 0.214, *P* = 0.031).In addition, a significant positive association between body fat and NEAP (*β* = 0.218, *P* = 0.001) and BAI and NEAP (*β* = 0.182, P = 0.037) was found after controlling for confounders.

A statistically significant inverse association was observed between TBW and NEAP (*β* = − 0.217, *P* = 0.001), and the percentage of FFM and NEAP (*β* = − 0.229, *P* = 0.001).

The linear regression analysis was used to examine the association between fat mass and NEAP and PRAL scores separately in men and women. Based on the results, in the adjusted model, there was no statistically significant association between fat mass and NEAP score among men (*β* = 0.025, *P* = 0.865; 95%CI − 0.058; 0.069), while there was a significant positive association between fat mass and NEAP score among women (*β* = 0.518, *P* < 0.0001; 95%CI 0.058; 0.149). Furthermore, in the adjusted model there was no statistically significant association between fat mass and PRAL score among men (*β* = 0.056, *P* = 0.759; 95%CI − 0.082; 0.112), and women (*β* = 0.142, *P* = 0.270; 95%CI − 0.035; 0.124).

In addition, the linear regression analysis was used to examine the association between fat-free mass, and NEAP and PRAL score separately in men and women. In the adjusted model, there was a positive association between fat-free mass and NEAP score among men (*β* = 0.292, *P* = 0.037; 95%CI 0.005; 0.150), while there was no significant association between fat-free mass and NEAP score among women (*β* = − 0.148, *P* = 0.292; 95%CI − 0.063; 0.019). Also, in the adjusted model, there was no statistically significant association between fat-free mass and PRAL score among men (*β* = 0.316, *P* = 0.071; 95%CI − 0.009; 0.213), and women (*β* = 0.074, *P* = 0.605; 95%CI − 0.049; 0.084).

## Discussion

This cross-sectional study included 207 physical education students and found a significant positive association between a high DAL (NEAP score) and anthropometric indices, including hip circumference, BMI, fat mass, and BAI. Also, a significant inverse association was observed between a high DAL (NEAP score) and fat-free mass and TBW. There was no significant association between the PRAL score and anthropometric indices and body composition after controlling for confounders.

DAL estimates the effect of food intake on acid–base status using two NEAP and PRAL methods. A higher NEAP and PRAL score imply a higher acidity of the diet. A higher intake of dairy products and meats is related to a higher DAL, while a higher intake of vegetables and fruits is related to a lower DAL [23, 24]. Regarding NEAP and PRAL scores, the results of our study have shown a higher acidity of diet than other studies on Iranian adults [23, 25]. This difference can be justified by the higher physical activity of participants. As mentioned before, physical exercise and sports lead to a higher production of organic acids in the body [15]. Moderate and high-intensity exercise alters the acid–base balance and causes rapid muscle fatigue and metabolic changes [15, 26]. Several studies reported that the NEAP score of young individuals was about 50 mEq/day [22, 27]. However, a study found that the average NEAP score of aerobic and anaerobic athletes was slightly higher than normal individuals (53.7 vs 56.9 mEq/day) [28]. Furthermore, the average NEAP score of Lithuanian high-performance athletes was 126.1 mEq/day, which was higher than 100 mEq/day [13].

Diet plays an important key role in metabolic changes in the body. Previous studies revealed that a low-carbohydrate, high-protein diet leads to a high NEAP [28, 29]. While there was no association between carbohydrate intake and NEAP or PRAL in the present study, protein consumption was higher in the third tertile of NEAP and PRAL. Studies have revealed that excessive protein intake causes cortisol secretion and hypercortisolism which leads to proteolysis by itself [30, 31]. Although we found no significant association between muscle mass and DAL in this study, there was a significant negative association between FFM and NEAP. Also, a higher protein intake and higher DAL are associated with calcium excretion. Therefore, calcium intake is essential for prevention, particularly, in individuals with a high DAL [32]. Unfortunately, the average calcium intake in the participants of the current study was lower than the daily calcium recommendations (1202 vs. 1500 mg) [33].

Our results revealed a significant positive association between NEAP score and BMI. This finding is in line with Mozaffari et al. study [9], which found a significant positive association between NEAP and overweight and obesity (BMI > 25 kg/m^2^). A cross-sectional study among 375 Iranian adult women showed that DAL was directly associated with greater WC and waist-to-hip ratio [34]. Another cross-sectional study that included 5620 Iranian adults aged 20–70 years found a significant association between PRAL and a higher WC and weight [23]. However, our findings and results of a Japanese cross-sectional study that included women aged 18–22 years revealed no significant association between PRAL score and WC or obesity [35]. Other studies in adults aged 19–70 years showed a significant positive association between DAL and obesity in both genders [23, 24]. A randomized clinical trial revealed that DAL was positively correlated with changes in body weight, fat mass, and visceral fat [36]. Higher acidity and metabolic acidosis might increase proteolysis by increasing mRNA coding [37]. Also, DAL metabolic acidosis leads to impaired secretion of insulin-like growth factor 1, followed by an increase in insulin resistance [38]. Another possible explanation for the effect of DAL on anthropometric indices is the positive association between insulin resistance and serum markers of acidosis such as low bicarbonate, high anion gap and high lactate. Furthermore, it should be considered that insulin resistance increases the obesity risk. [38–40].

Investigating among 243 seniors revealed that DAL was negatively associated with total lean mass especially in women and probably an alkaline diet may be beneficial for preserving lean mass [41]. As mentioned previously, our findings showed an inverse association between DAL and FFM, which is in line with a cross-sectional study in women aged 18–79 years [12]. Welch et al. have indicated that more alkaline PRAL is positively associated with FFM, independent of age, physical activity, and smoking [12]. The high bicarbonate precursors in vegetables and fruits can induce metabolic alkaloids. A study on adults considered urinary potassium excretion as an indicator of intake of potassium and fruits and vegetables and reported a positive association between urinary potassium excretion and lean body mass in a study in adults [7]. Our study showed a strong significant inverse association between potassium intake and PRAL and NEAP. On the other hand, considering the high amount of sulfur-containing amino acids in the animal products, a higher intake of red meat and animal products leads to hydrogen sulfate and phosphoric acids from phospholipids and a higher DAL [11]. Most of the acidogenic diets induce proteolysis, amino acid excretion, and reduced protein synthesis cause muscle loss [7]. Different findings may be due to different ranges of DAL in various studies. Although previous studies are different in the sample size, race, gender, age, lifestyle, dietary habits, adjustments, and assessment tools, the evidence shows that DAL is related to obesity.

Although the exact mechanism of dietary acid load, which can affect body composition, is not completely known, studies revealed that protein and nutrients such as potassium and magnesium might affect body composition and anthropometric indices such as muscle mass [42, 43]. Furthermore, a higher intake of fruits and vegetables; thus, higher consumption of carotenoids and antioxidants (thereby lower DAL) may have a protective role against inflammation and oxidative stress as causes of obesity and metabolic disorders [44, 45].

To the best of our knowledge, this is the first study that examined the association between DAL and anthropometric indices in Physical Education students. Furthermore, individuals with comorbidities and chronic diseases were not included. However, several limitations should be considered. Firstly, the cross-sectional design cannot describe the causal effect. Secondly, using FFQ contributes to dietary misclassification. Thirdly, DAL was calculated using dietary intake estimation based on FFQ results which might under or overestimate the dietary intake. Fourthly, cortisol as an important biochemical factor related to obesity and DAL was not measured in this study. Fifthly, physical education students probably were more aware of their health and related factors, including physical activity and diet. This could be considered because these participants could have a higher level of physical activity, affecting the NEAP and PRAL scores. The body composition analysis results suggested that there is not a wide range of variation. Most of the participants are in normal categories in the present study thus, it will advance the knowledge if the comparison is made with obese individuals in future studies.

## Conclusion

In the present study, individuals with a higher DAL score may have a higher weight, HC, BMI and FM. There might be an association between DAL and obesity. However, more well-designed prospective studies are needed to confirm our findings.

## Data Availability

The data only will be available upon the reasonable request.
